# Low-stress and high-stress singing have contrasting effects on glucocorticoid response

**DOI:** 10.3389/fpsyg.2015.01242

**Published:** 2015-09-04

**Authors:** Daisy Fancourt, Lisa Aufegger, Aaron Williamon

**Affiliations:** Centre for Performance Science, Royal College of Music, London, UK

**Keywords:** music, singing, stress, glucocorticoids, cortisol, performance science

## Abstract

Performing music in public is widely recognized as a potentially stress-inducing activity. However, despite the interest in music performance as an acute psychosocial stressor, there has been relatively little research on the effects of public performance on the endocrine system. This study examined the impact of singing in a low-stress performance situation and a high-stress live concert on levels of glucocorticoids (cortisol and cortisone) in 15 professional singers. The results showed a significant decrease in both cortisol and cortisone across the low-stress condition, suggesting that singing in itself is a stress-reducing (and possibly health-promoting) activity, but significant increases across the high-stress condition. This is the first study to demonstrate that singing affects cortisol as well as cortisone responses and that these responses are modulated by the conditions of performance.

## Introduction

Live music performance can be highly stress-inducing, typically involving the simultaneous coordination and execution of complex cognitive, perceptual, motor and social skills in front of an audience. While performance stress has been studied in music psychology for several decades (Kenny, 2011; see Brugués, 2011; Sârbescu and Dorgo, 2014; Kenny and Ackermann, 2015), corresponding research into the *physical* effects of performing music by comparison is meager. Preliminary studies have demonstrated significant increases in heart rate and systolic and diastolic blood pressure in response to high-stress performance conditions (Craske and Craig, 1984; Abel and Larkin, 1990; Brotons, 1994). More recent research has shown that live performance can lead to a loss of complexity in cardiovascular response, a state indicative of difficulty in physically adapting to stressful conditions (Williamon et al., 2013). It is noteworthy that this is found even at the highest of profession levels where regular public performance is commonplace.

Within the field of psychobiology, acute psychosocial stress has been shown to have wide ranging effects across the endocrine system. One of the major pathways to be activated during acute stress is the hypothalamic-pituitary-adrenal (HPA) axis. Corticotropin releasing hormone (CRH) secretion in the hypothalamus in response to stress leads to the release of adrenocorticotropic releasing hormone (ACTH) from the pituitary gland. This in turn stimulates glucocorticoids within the adrenal cortex, including cortisol, a steroid hormone that causes glucogenesis, suppresses the immune system and aids in metabolism, and cortisone, a downstream metabolite of cortisol and another steroid hormone that also suppresses the immune system (Smith and Vale, 2006; Frodl and O’Keane, 2013). Increased HPA activity in response to stressful situations is part of the natural fight-or-flight response and leads to increases in glucose, inhibited insulin production, and narrowing of arteries and occurs alongside increases in epinephrine leading to raised blood pressure (Puglisi-Allegra, 1990; Schneiderman et al., 2005). However, a decrease over time in the cortisol/cortisone ratio has been shown in multiple studies to be a relaxation response and to occur alongside reductions in stress level (Vogeser et al., 2001, 2003).

Despite music performance being a particularly apt domain for studying the effects of psychosocial stress, there has been little research into its effects on HPA activity. Looking at instrumentalists, Fredrikson and Gunnarsson (1992) examined psychobiological changes in string players in the absence and presence of an audience and found that greater levels of cortisol were released in public performance situations compared with private playing. These changes were found independently of whether the musicians reported themselves as “high-anxious” or “low-anxious”, suggesting that situational factors (i.e., public high-stress vs private low-stress) rather than individual differences were responsible for the results. However, the team analyzed single samples rather than comparing pre- and post-performance levels, and so the exact pattern of change in cortisol response *across* each performance could be not determined. Gill et al. (2006) measured cortisol prior to low- and high-stress conditions. They found higher levels prior to a high-stress performance in front of an audience compared with a low-stress performance without an audience. Again, however, there was no analysis of the change in cortisol within each condition. From an examination of group orchestral performances, two studies have found that cortisol levels were significantly higher across the diurnal cycle on the day of a high-stress concert than on a non-performance day (Halleland et al., 2009; Pilger et al., 2014).

With regard to singing, there has been even less research to date. Beck et al. (2000) showed that singing in low-stress conditions led to reductions in cortisol, while high-stress singing led to increases (Beck et al., 2000). However, this study compared responses to low- and high-stress conditions in isolation from one another and did not analyze for differences between conditions or within individuals, focusing on correlations alone. Given the different physical demands of playing an instrument versus singing and that instrumentalists and singers experience differing occupational stressors in their professional lives (Eller et al., 1992), one cannot assume that psychosocial stress is manifested in the same way in both groups. Consequently, more research into how stress is experienced by singers is warranted.

Our study aimed to build upon previous research by examining changes in stress hormone levels in singers between and within low- and high-stress performance situations. In order to confirm involvement of the adrenal cortex, we examined cortisol and the additional hormone cortisone, another glucocorticoid secreted in response to HPA activity. Based on the available research with instrumentalists and singers, we hypothesized that singing in high-stress conditions would lead to an increase in glucocorticoid response. However, taking a cue from the arts-in-health literature, where singing has been shown generally to be a health-promoting activity (Clift and Hancox, 2001), we predicted that singing in low-stress conditions would lead to reductions in glucocorticoid response as well as a decrease in the cortisol/cortisone ratio, indicative of a relaxation response.

## Materials and Methods

### Participants

Fifteen professional singers (from a choir of 19) volunteered to take part in the study: 6 men and 9 women, with a mean age of 32.1 years (SD = 5.6, range 25–45). Participants had an average of 21.1 years of musical experience (SD = 4.7, range 12–28), and in the previous 12 months, they had performed an average of 39.1 times in public (SD = 27.4). Exclusion criteria included the current taking of immunosuppressive or hormone medication or pregnancy, as this was anticipated to affect hormone levels. The research was granted ethical approval by the Conservatoires UK Research Ethics Committee and was conducted according to ethical guidelines of the British Psychological Society. Informed consent was obtained from all participants, and no payment was given in exchange for participation.

### Procedure

The singers were monitored during performances of a program of compositions by Eric Whitacre on two consecutive evenings at the London venue Union Chapel. The performances were scheduled to take place between 19:30 and 20:30 to minimize the effects of diurnal hormone cycles. For each, participants were asked to arrive 30 min beforehand; after giving their first saliva sample, they waited quietly without eating or drinking, except for small sips of water to help their voices. Immediately before and after performing, they completed questionnaires and provided a saliva sample (see “State anxiety” and “Glucocorticoids”). On the first evening, they sang in low-stress conditions (i.e., without an audience) led by the choir’s principal conductor, and on the second evening, they sang the same program, with the same conductor, in a live concert given in front of a paying audience of 610 people. Due to the nature of live concerts, in which an interval is customary, we collected all post-singing data (state anxiety and saliva samples) during the interval rather than at the end of the whole concert, particularly to avoid the effects of confounding variables, such as physical activity and talking, on endocrine response during the interval period. Consequently, it was not possible in this study to measure stress recovery, and as such, we have looked purely at stress reactivity.

### State Anxiety

In order to confirm that the two conditions corresponded to genuinely low- and high-stress conditions, participants were asked to complete Form Y1 (state anxiety) of the State-Trait Anxiety Inventory (Spielberger et al., 1983). This 20-item questionnaire measures a person’s temporary emotional state, consisting of subjective feelings of nervousness and perceived activation and arousal. Each item is rated on a 4-point scale (1 = “almost never” to 4 = “almost always”), and the cumulative score ranges from 20 (low state anxiety) to 80 (high). For comparison with the results presented below, moderate levels of anxiety are represented by scores of 36.47 (SD = 10.02) for young men and 38.76 (SD = 11.95) for young women (Spielberger et al., 1983).

### Glucocorticoids

Saliva samples were collected via a passive drool method facilitated by polypropylene straws into low-bind polypropylene 2 mL cryovials (Eppendorf, UK) immediately before and after singing (i.e., at 19:30 and 20:30 on both days). Samples were stored at –20°C for a period of 3 weeks prior to analysis using high performance liquid chromatography-tandem mass spectrometry (LC-MS/MS) with Atmospheric Pressure Chemical Ionization (APCI) coupled with on-line solid phase extraction (SPE; Gao et al., in press; Deuterated internal standard samples, Toronto Research Chemicals Inc, Canada; LC-MS grade methanol, Fisher Chemicals, UK). The glucocorticoids analyzed were cortisol and cortisone, stress hormones released from the adrenal cortex that play a crucial role in immune, metabolic, cardiovascular and homeostatic functions. Here, concentrations are given as ng/ml (cortisone) and nmol/l (cortisol). Cortisol quantities were converted to ng/ml for the cortisol/cortisone ratio.

### Data Preparation and Analysis

An *a priori* sample size calculation looking for a large effect size (0.5), an alpha of 0.05, and power of 0.9 revealed that 10 participants were needed for the study. To allow for drop-outs and non-analysable samples, fifteen participants were recruited to the study. Statistical analyses were performed using SPSS (Version 22.0, SPSS Inc., USA). The distributions of both glucocorticoids were positively skewed, and so the data were logarithmically transformed. Differences in baseline levels of glucocorticoids prior to the low- and high-stress conditions were analyzed using paired-samples *t*-tests. We subsequently used 2 × 2 repeated measures analyses of variance (ANOVAs) to test for changes in state anxiety, cortisol and cortisone, across time (pre- vs post-performance), between conditions (low- vs high-stress situations), and their interaction. Cortisol/cortisone ratios were calculated by dividing cortisol values (converted to ng/ml) by cortisone values (ng/ml), with a decrease in the cortisol/cortisone ratio indicating a decrease in stress response. The ratio data did not meet requirements for parametric statistical tests, and so changes across time were analyzed for each condition using Wilcoxon signed-rank test, the non-parametric equivalent of the paired samples *t*-test. Some samples were unsuitable for analysis or levels fell below the limit of detection, and so these were excluded, thereby accounting for the degrees of freedom reported. We assessed correlations between state anxiety and each glucocorticoid with Pearson’s product-moment correlation coefficient.

## Results

**FIGURE 1:**
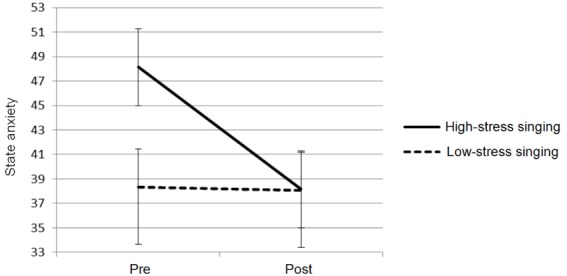
**Mean responses and standard error for state anxiety for singing in low- and high-stress performance conditions**.

State anxiety scores revealed no significant effect of time, but they did show a significant overall difference in anxiety between the low- and high-stress conditions (*F*_1,11_ = 7.569, *p* < 0.05). Furthermore, there was a significant time × condition interaction (*F*_1,11_ = 5.422, *p* < 0.05), with perceived anxiety levels in the high-stress condition particularly high before performance and then steeply declining afterward, yet no discernible change across the low-stress condition (see Figure 1). These findings confirm that the two performance conditions varied in the intensity of stress (low vs high) and that the difference in stress levels directly impacted on singers’ perceived anxiety.

**FIGURE 2:**
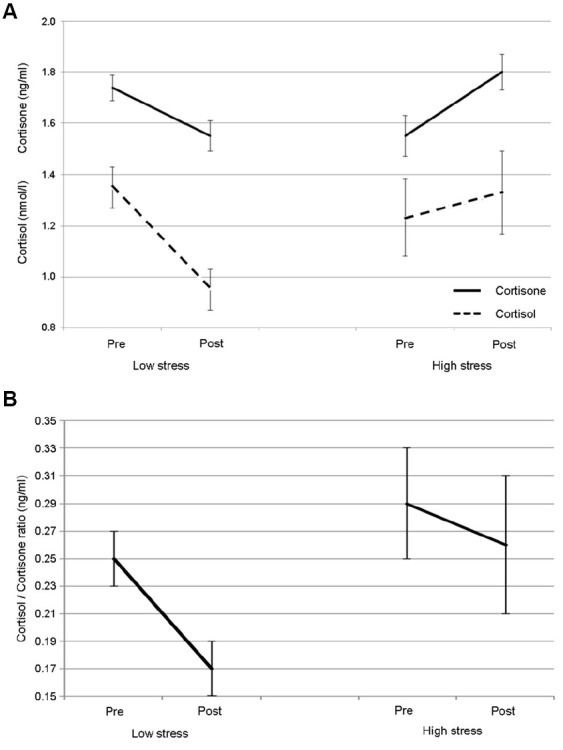
**Mean responses and standard error for (A) the glucocorticoids cortisol and cortisone and (B) the cortisol/cortisone ratio for singing in low- and high-stress performance conditions**.

Preliminary analyses showed no significant differences in the first measurement (pre-) between the two conditions in cortisol or the cortisol/cortisone ratio. However, levels of cortisone were significantly higher prior to the high-stress condition (*t*_13_ = 2.385, *p* < 0.05). Analyses of cortisol and cortisone revealed that there were no significant effects of time (pre- vs post-performance) or condition (low- vs high-stress), but there was a significant time × condition interaction: cortisol *F*_1,13_ = 6.712, *p* < 0.05; cortisone *F*_1,13_ = 15.813, *p* < 0.005. Figure 2A shows a drop in cortisol and cortisone across the low-stress performance and conversely an increase in both hormones across the high-stress performance. For the cortisol/cortisone ratio, for which higher values are associated with higher stress levels, decreases were seen across both conditions, with a significant effect of time for the low-stress performance (*z* = –2.953, *p* < 0.01) but not for the high-stress performance. As shown in Figure 2B, while the ratio decreased across both performances, it was overall lower for the low-stress condition and decreased more sharply over time than in the high-stress condition. These changes in glucocorticoid response were independent of age, sex, number of years’ singing experience, and the number of concerts already performed that year. We found no significant correlations between state anxiety and changes in cortisol, cortisone or the cortisol/cortisone ratio; however, due to the lag time in glucocorticoid production, a correlation was not anticipated.

## Discussion

This study demonstrated that singing overall was associated with a reduced cortisol/cortisone ratio, indicating a decrease in overall stress response. However, across the low-stress and high-stress performances, singing was associated with contrasting glucocorticoid responses: low-stress conditions showed decreases in cortisol and cortisone, while high-stress conditions showed increases in both.

These results confirm previous findings that low-stress performance conditions lead to reductions in cortisol and that high-stress conditions lead to increases. They extend them by showing that cortisone too is affected, which is indicative of a more general HPA axis response (Beck et al., 2000). However, studies have shown that this HPA axis response is modulated by the specific way a person appraises a stressful situation, and the degree to which the stress response is seen is affected by how well a person is able to cope with the psychological perception of the stressor (Kemeny, 2003). Consequently, the evidence of increased glucocorticoids in response to high-stress singing, with no significant relationship to experience, age or number of previous concerts, demonstrates that despite persistent exposure to performance situations, singers still exhibit a rather basic fight or flight stress response. This highlights the need to explore a wider range of performance preparation strategies and training interventions for singers than is currently available (Williamon, 2004; Williamon et al., 2014). Equally noteworthy is the decrease that we found in low-stress conditions. Research has questioned whether the professionalization of music militates against its relaxing and potentially therapeutic effects (Wynn Parry, 2004; Kenny, 2011). However, there is evidence here that singing by professionals under low-stress conditions can still lead to reductions in biological stress response.

Previous studies have shown similarities in psychological and biological response, such as higher reported anxiety alongside higher cortisol levels (Gill et al., 2006). In this study, despite the difference in baseline anxiety levels between the low- and high-stress conditions, there were no significant baseline differences in cortisol between the conditions, but cortisone levels were significantly higher. While this does attest to there being *some* parity between psychological and biological responses, the state anxiety scores and cortisone levels were not significantly correlated and there were also interesting discrepancies across time. Regarding the lack of correlation, this was expected as lag times in glucocorticoid production mean that change patterns in such measures do not typically coincide statistically (Allen et al., 2014). As for other discrepancies, there were no reductions in state anxiety, for instance, despite the reduction in glucocorticoids during the low-stress condition. As anxiety levels before and after the low-stress condition were nearly identical to those after the high-stress condition, one possible explanation of the absence of a reduction in reported anxiety is that participants achieved a personal “floor” effect. This would suggest that, although singing was able to reduce psychological anxiety when participants reached higher than their usual levels, it was not able to reduce perceived anxiety when at comparatively low levels.

Confirmation of the changes in glucocorticoid response to singing raises questions as to how such changes impact on wider biological response. There is a strong literature exploring interactions between glucocorticoids and components of the immune system including white blood cells and cytokines (Petrovsky et al., 1998; Wiegers and Reul, 1998; Miller et al., 2002; Kunz-Ebrecht et al., 2003). Indeed, Pilger et al. (2014) showed that orchestral performance both modulated cortisol levels and also increased levels of two biomarkers of the immune system: the cytokine Interleukin 6 and the peroxidase enzyme myeloperoxidase (Pilger et al., 2014). A recent study of group drumming showed that music making can modulate levels of both glucocorticoids and cytokines (Fancourt et al., in press). To explore the implications of the changes in glucocorticoids noted here, future studies should elucidate whether similar alterations occur in immune biomarkers, and as such, whether low- or high-stress performance conditions could modulate immune response.

Naturally, there are limitations to this study. The sample size was small (although sufficient for the reported analyses) and somewhat homogenous in age. Consequently, it is not known how hormone levels would alter in older singers. The study also used a fairly homogenous style of music (i.e., calm, classical), and the effects of different genres are unknown. Further research is needed to ascertain how responses differ across other populations and conditions. Finally, all participants took part in the low-stress condition prior to the high-stress condition. This lack of counterbalancing may have affected our results, for example by creating a novelty effect the first time samples were taken that may have been responsible for why cortisol levels were equally as high in the first low-stress saliva sample compared with the first high-stress sample (see Figure 2). Future studies should explore the effects of reversing these conditions.

## Conclusion

This study demonstrates for the first time that singing can affect both cortisol and cortisone responses and that these responses are modulated by the conditions of performance. This shows that the act of performing not only has an effect on the psychological and physiological states of singers but also alters their biological activity. Furthermore, this study demonstrates that low-stress performances are not neutral, but they too can affect biological responses. Future studies should consider the implications for these endocrine changes on wider immune and neurological function and also examine whether protective strategies for managing music performance anxiety can reduce some of the negative consequences of steroid hormone release.

## Author Contributions

All authors contributed extensively to the work presented in this paper.

### Conflict of Interest Statement

The authors declare that the research was conducted in the absence of any commercial or financial relationships that could be construed as a potential conflict of interest.
